# Salt-induced 7-deoxypactamycin and oligomycin production in *Streptomyces* sp. strain TUA-HK1GM isolated from kusaya gravy

**DOI:** 10.1038/s41429-026-00934-y

**Published:** 2026-06-09

**Authors:** Sachiko Masaki, Sho Ogaki, Nana Kanayama, Asahi Hirata, Aiko Teshima, Khaled H. Almabruk, Takahiro Osada, Ryosuke Unno, Morio Ishikawa, Taifo Mahmud, Kenji Arakawa, Toshihiro Suzuki

**Affiliations:** 1 Department of Fermentation Science and Technology, Graduate School of Applied Bioscience, Tokyo University of Agriculture, 1-1-1 Sakuragaoka, Setagaya, Tokyo 156-8502 Japan; 2 Graduate School of Integrated Science for Life, Hiroshima University, 1-3-1 Kagamiyama, Higashi-Hiroshima, Hiroshima 739-8530 Japan; 3 Department of Fermentation Science, Faculty of Applied Bioscience, Tokyo University of Agriculture, 1-1-1 Sakuragaoka, Setagaya, Tokyo 156-8502 Japan; 4 Department of Pharmaceutical Sciences, Oregon State University, Corvallis, OR 97331, 541-737-1000 USA; 5 Osada Shouten, 789 Mitsune, Hachijojima, Tokyo 100-1511 Japan

**Keywords:** Natural product synthesis, Drug discovery and development

## Abstract

*Streptomyces* produce various antibiotics and bioactive compounds as secondary metabolites. Although they are normally found in soil, strains of *Streptomyces* have also been discovered in non-soil environments, including marine habitats. These non-terrestrial strains may have specific metabolic features compared with those of soil-dwelling *Streptomyces*. In this study, we investigated phenotypic and taxonomic characteristics, and antibiotic production of a *Streptomyces* sp. strain TUA-HK1GM that we previously isolated from kusaya gravy, a fermented fish gravy high in salt content. Microbiological analysis revealed distinct properties compared to closely related species, particularly in its ability to grow at 7% NaCl. Phylogenetic analysis suggested that strain TUA-HK1GM is a novel actinomycete species. We investigated the effects of NaCl supplementation on secondary metabolite biosynthesis, with a focus on antibiotic production. This strain produced antifungal compounds in the absence of NaCl, and these compounds were identified as oligomycins A and B. On the other hand, a large amount of anti-*Staphylococcus* compound was produced alongside oligomycin in the presence of NaCl. This compound had *m/z* values of 543.2813 [M + H]⁺ and 565.2633 [M+Na]⁺, which were consistent with those of 7-deoxypactamycin. Enhancement (32-fold) of oligomycin A production was also observed in a manner dependent on the duration of NaCl exposure. Supplementation with NaCl during cultivation demonstrated that prolonged exposure to NaCl increased the antibiotic production. These results indicate that the novel strain TUA-HK1GM has a characteristic mechanism that involves secondary metabolic activation by NaCl.

## Introduction

*Streptomyces* are Gram-positive bacteria that produce a wide variety of antibiotics and bioactive compounds important to the medical, pharmaceutical and agricultural industries. Most *Streptomyces* species are isolated from soil environments, but the rate of discovery of novel *Streptomyces* in soil and the antibiotics they produce has decreased [1, 2]. Therefore, interest has increased considerably in the isolation of novel and characteristic Actinomycetes from environments other than soils, such as the ocean [3]. We have focused on kusaya gravy, the fermented brine used in the manufacture of the aquatic fermented fish food, kusaya, as a new source for the isolation of Actinomycetes and antibiotics.

Kusaya, a traditional Japanese fermented fish product from the Izu Islands is manufactured by soaking raw fish in kusaya gravy, a fermented brine that has been reused and maintained for over a century [4–7]. A distinguishing feature of kusaya manufacturing is the open fermentation system in which no specific microorganisms are artificially inoculated [8, 9]. Thus, kusaya gravy contains diverse microbiota derived from raw fish and seawater, forming a distinctive microbial ecosystem [9]. Moreover, kusaya gravy functions as *quasi*-concentrated seawater enriched with organic components from both fish and seawater, that is expected to contain numerous potentially novel marine-derived microorganisms [9, 10]. One notable characteristic of kusaya is high preservability compared to other salted-dried fish [11], which has traditionally been attributed to the antimicrobial substances produced by microorganisms in kusaya gravy that inhibit moulds, yeasts, and spoilage bacteria. In fact, spoilage bacteria have not been detected in metagenomic analyses of kusaya gravy in recent years [9, 12].

We previously demonstrated that kusaya gravy exhibits direct antimicrobial effects, showing strong activity against various test microorganisms, especially moulds [10]. Even when the gravy was extracted with organic solvents, antimicrobial activity was detected [10], indicating that multiple antimicrobial compounds coexist in the gravy. Given the indication of the presence of antibiotic-producing bacteria in kusaya gravy, we attempted to isolate antibiotic-producing actinomycetes and obtained several *Streptomyces* spp., including strains TUA-HKG01W, TUA-HKG03B, TUA-KBG03S, TUA-HMG27, and TUA-HK1GM [10]. These strains were found to produce substances with strong antimicrobial activity against moulds, yeasts, and various bacteria. Among these isolates, strain TUA-HK1GM exhibited potent antimicrobial activity against *Aspergillus* and *Fusarium* [10]. However, the microbiological characteristics of this strain and details of its antimicrobial compounds remain unclear. As kusaya gravy represents a unique ecological niche, the strain TUA-HK1GM isolated from it may exhibit characteristics specifically influenced by this environment.

In this study, we aimed to better understand the specific characteristics of strain TUA-HK1GM. We also investigated the antimicrobial compounds that the strain produces and the effects of NaCl concentration on its growth and antibiotic production.

## Material and methods

### Bacterial strains and culture media

*Streptomyces* sp. strain TUA-HK1GM was isolated from Hachijojima’s kusaya gravy described in our previous report [10]. In the present study, the strain was grown in ISP2 medium containing 4.0 g glucose, 4.0 g yeast extract (Oxoid, Basingstoke, UK), and 10 g malt extract (Oxoid) to a volume of 1 l (pH 7.3) or ISP2 medium containing 3% NaCl (ISP2-3N). *Aspergillus brasiliensis* NBRC 9455 and *Staphylococcus epidermidis* NBRC 12993 were used for antimicrobial assay using paper disk assays according to Takeuchi et al. [7]. Both test strains were grown in TSB medium containing 30 g trypticase soy broth (BD/Difco, Sparks, MD, USA) to a volume of 1 l. Unless otherwise specified, all reagents were obtained from Wako Pure Chemical Industries, Ltd. (Osaka, Japan).

### Morphological, physiological, and biochemical characteristics

Growth characteristics (growth, colour of aerial and substrate mycelia, formation of spores, soluble pigment, and production of melanoid pigments) of strain TUA-HK1GM in various media (ISP2–7) were investigated following incubation at 30 °C for 7–21 days. The morphology of the colony and spore-bearing hyphae with spore chains was observed under a stereomicroscope (SMZ; Nikon, Tokyo, Japan) and a light microscope (BX50F4; Olympus, Tokyo, Japan), respectively. Gelatin liquefaction, starch hydrolysis, nitrate reduction, and milk peptonisation were examined, as described by Waksman [13]. Carbon source utilisation was tested following growth on Pridham and Gottlieb’s medium (ISP9 medium) containing a carbon source [14]. Sodium chloride tolerance was investigated according to Tresner et al. [15] using ISP2 medium. The plates were incubated at 30 °C for 7–21 days. All tests, except for NaCl tolerance, were performed by TechnoSuruga Laboratory Co., Ltd.

### Purification of antimicrobial compounds

For the identification of antifungal compounds, the strain TUA-HK1GM was cultured in ISP2 liquid medium at 28 °C with shaking for 10 days. The fermented culture broth (2.2 l) was then centrifuged at 10,000 ×*g* for 10 min. The supernatant was extracted three times with the same volume of ethyl acetate, and the organic phase was dried over Na_2_SO_4_, filtered, and concentrated *in vacuo*. The extracts were dissolved in methanol, and aliquots were analysed using thin layer chromatography (TLC) and TLC-bioautography using a mixture of hexane-ethyl acetate (2:3, v/v). The crude extract (59.2 mg) was subjected to silica gel (60 N, 20 g; column size: 15 mm i.d. × 20 cm; Kanto Chemical, Tokyo, Japan) column chromatography, and the antifungal compounds were eluted with only *n*-hexane-ethyl acetate (2:3, v/v) to yield 4.7 mg of the purified active fraction.

For the identification of antibacterial compounds, strain TUA-HK1GM was cultured in ISP2-3N liquid medium at 28 °C with shaking at 121 rpm for 10 days. Crude extracts (560 mg) were obtained from the culture broth (4.0 L) in the same way as described above, and aliquots were analysed using TLC and TLC-bioautography using a mixture of chloroform–methanol (9:1, v/v) and butanol-acetic acid-H_2_O (20:1:1, v/v). The crude extract was subjected to gel-filtration chromatography using Sephadex LH-20 columns (14 g; column size: 15.4 mm i.d. × 34 cm; GE Healthcare BioScience AB, Uppsala, Sweden), and fractions were eluted with methanol. The fraction with antimicrobial activity was further purified through silica gel column chromatography (60 N, 5 g; column size: 10 mm i.d. × 13 cm), eluting with chloroform-methanol (4:1 v/v). The active fractions were then subjected to another silica gel column (60 N, 4 g; column size: 10 mm i.d. × 10 cm) and further purified with butanol-acetic acid-H_2_O (20:1:1, v/v) to yield 0.1 mg of the final purified active compound.

### Structural elucidation of antimicrobial compounds

To elucidate their structure, each purified antimicrobial compound was subjected to electrospray ionisation-mass spectrometry (ESI-MS) using an LTQ-Orbitrap XL mass spectrometer (Thermo Fisher Scientific, Waltham, MA, USA). Nuclear magnetic resonance (NMR) spectra of antifungal compounds were recorded on a JEOL ECA-500 spectrometer equipped with a field gradient accessory (JEOL Ltd., Tokyo, Japan). NMR chemical shifts were recorded as *δ* values (ppm). The coupling constants in the ^1^H NMR spectra were shown as *J* values (Hz). Chloroform-*d*_1_ (99.8 atom %D, containing 0.03% tetramethylsilane TMS; Kanto Chemical) was used as the solvent for ^1^H and ^13^C NMR.

Comparative LC-MS and ESI-MS/MS analyses for antimicrobial compounds were performed on a Xevo G2-XS LC-quadrupole time-of-flight mass spectrometer (QToF-MS) (Waters Corporation, Manchester, UK) using a COSMOSIL Cholester C_18_ reverse-phase chromatography column (250 mm×ϕ4.6 mm, Nacalai Tesque, Kyoto, Japan). The mobile phases were (A) water and (B) acetonitrile, both with 0.1% formic acid, eluted at a flow rate of 0.3 ml/min at 40 °C. The mobile phase gradient was: 1 min, 25% B; 10 min, 45% B; 15 min, 45%; 20 min, 25% B; and 30 min, 25% B. Injection volume was 3 μl. The effluents were monitored by extracted ion chromatograms at *m/z* 543.28.

### Genome sequencing and genome characterisation

Genomic DNA was extracted from strain TUA-HK1GM cultured in ISP2 medium using NucleoBond AXG columns and NucleoBond Buffer Set III (MACHEREY-NAGEL GmbH & Co.KG, Nordrhein-Westfalen, Germany). Genome sequencing was performed using the PacBio Sequel II system (Pacific Bioscience, Menlo Park, CA, USA) and annotated using the DDBJ Fast Annotation and Submission Tool **(**DFAST**)**. Genome-based phylogenetic analysis and digital DNA–DNA hybridisation (dDDH) were performed using the Type (Strain) Genome Server (TYGS), and orthologous average nucleotide identity (ANI) analysis was conducted using OrthoANI [16], based on the assembled genome.

To assess the number of biosynthetic gene clusters (BGCs) for secondary metabolites and to confirm the presence of the oligomycin (*olm*) and 7-deoxypractamycin (*ptm*) BGCs on the chromosome of strain TUA-HK1GM, putative secondary metabolite BGCs were detected and analyzed using antiSMASH v7.0.1 [17].

### Effect of NaCl addition on antimicrobial compound production

Growth in the presence or absence of NaCl was investigated by measuring the dry cell weight (DCW) of 100 ml of culture broth. Culture broths of ISP2 and ISP2-3N media were filtered through filter paper (No. 2, Φ90 mm; Advantec, Tokyo, Japan) using an aspirator (AS ONE, Osaka, Japan) to separate the mycelia from the filtrates. The pellets on the filter were dried in a SI401 sterilisation ovens (Yamato Scientific, Tokyo, Japan) at 105 °C for 3 h, after which the DCW were measured. The filtrates were extracted with ethyl acetate, concentrated to 500 μl and 1 ml, respectively, depending on the DCW of ISP2 and ISP2-3N cultures, and used for antimicrobial assays.

The productivity of antimicrobial compounds was analysed on a Xevo G2-XS LC-QToF-MS using a COSMOSIL Cholester C_18_ reverse-phase chromatography column (250 mm×ϕ4.6 mm, Nacalai Tesque). The mobile phases were (A) water and (B) acetonitrile, both with 0.1% formic acid, delivered at a flow rate of 0.3 ml/min at 40 °C. The mobile phase gradient was: 1 min, 25% B; 10 min, 45% B; 18 min, 45%; 30 min, 90% B; 45 min, 90% B; 50 min, 25% B; and 60 min, 25% B. Injection volume was 3 μl. The eluates were monitored at an absorbance of 220 nm using a photodiode array eγ detector for oligomycins A and B, or by extracted ion chromatograms at *m/z* 543.28 for 7-deoxypactamycin. A mixture of oligomycin A, B, and C (1 mg/ml; MP Biomedicals, Santa Ana, CA, USA) was used as the standard for oligomycin and purified 7-deoxypactamycin served as the standard for 7-deoxypactamycin. To validate the analytical results, it was confirmed that the presence of 3% NaCl in the culture medium did not enhance extraction efficiency. This was verified by demonstrating that the levels of oligomycin B and trace amounts of 7-deoxypactamycin and oligomycin A detected in extracts from cultures grown in ISP2 medium were equivalent to those from identical cultures to which 3% NaCl was added only at the time of extraction.

To investigate the effect of NaCl in more detail, NaCl was added during cultivation, and the growth and antibiotic productivity were measured. NaCl was added to ISP2 medium cultures on day 4 (exposed to NaCl for 7 days) or 7 (exposed to NaCl for 4 days) to a final concentration of 3% (w/v), and incubation was continued for 10 days. ISP2 medium without NaCl (no exposure) and ISP2-3N (exposed to NaCl for 10 days) medium were also used for cultivation. After cultivation, the DCW were measured using the method described above. Antimicrobial compounds were extracted from the culture filtrates, concentrated based on the differences in DCW, and their antimicrobial activities were evaluated using antimicrobial assays and the compounds were quantified using extracted ion chromatograms and photodiode array eγ detector of the abovementioned LC-QToF-MS analysis.

### GenBank accession number

The genome sequence of strain TUA-HK1GM was obtained using PacBio Sequel II and deposited at DDBJ/EMBL/Genbank under (accession number Bioproject: PRJDB37698, Biosample: SAMD01693270).

## Result

### Phenotypic characteristics and phylogenetic position of strain TUA-HK1GM

We investigated the phenotypic characteristics and taxonomic position of strain TUA-HK1GM. The specific features of this strain are shown in Fig. S1 and Tables S1 and S2. The physiological and biochemical characteristics of this strain differed from those of *Streptomyces griseoruber* DSM 40281 ^T^, the closest relative based on 16S rRNA gene sequence analysis [10, 18]. Notably, this strain exhibited significant differences in NaCl tolerance and was able to grow even in the presence of 7% NaCl.

The whole-genome nucleotide sequence of strain TUA-HK1GM was determined to be 11,101,742 bp in length with a G + C content of 71.8%. Annotation using DFAST revealed that the genome contains 9,875 protein-coding sequences, 18 rRNA genes, 90 tRNA genes, and 3 CRISPR genes. A total of 35 secondary metabolite BGCs were detected using antiSMASH (Table S3). The Type Strain Genome Server (TYGS) analysis showed that the species most closely related to strain TUA-HK1GM was *S. griseoruber* DSM 40281 ^T^ [18] (Fig. S2). However, the digital DNA-DNA hybridisation and average nucleotide identity values were 54.3% and 92%, respectively, both below the index values of 70% and 95–96% typically used for species identification. These results indicated that strain TUA-HK1GM is a novel *Streptomyces* species.

### Structural elucidation of antifungal compounds

**Fig. 1:**
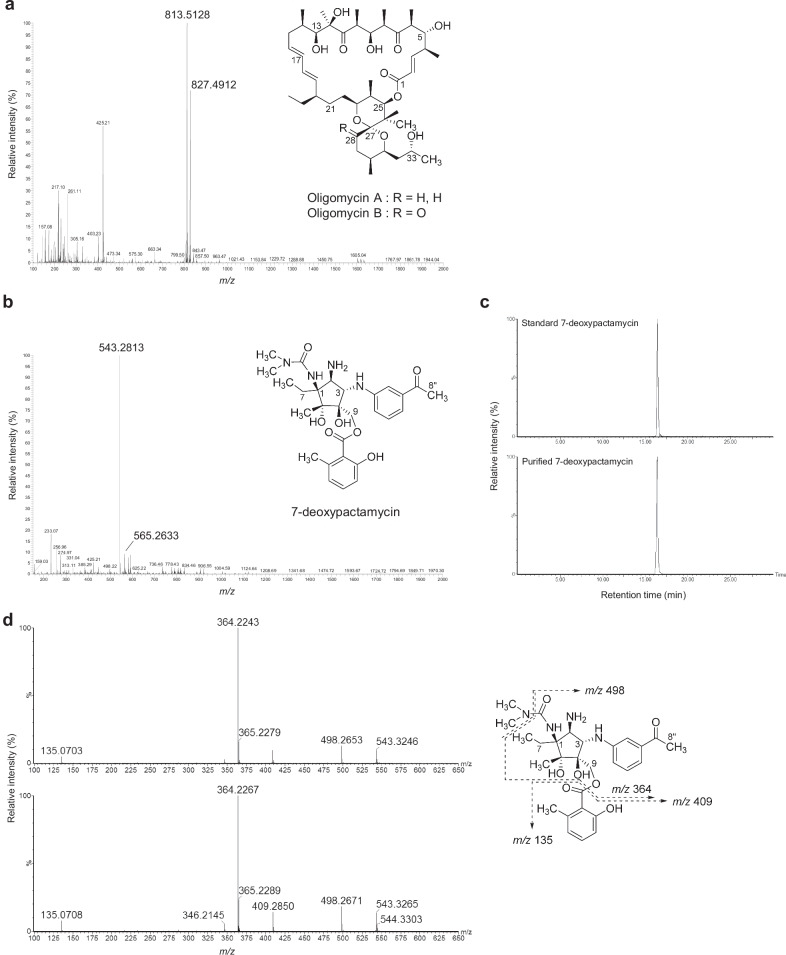
Structure elucidation of antimicrobial compounds. **a** Electrospray-ionisation-mass spectra and structures of antifungal compounds (oligomycins A and B). **b** Electrospray-ionisation-mass spectra and structures of antibacterial compounds (7-deoxypactamycin). **c** Comparative LC-MS analysis. **d** ESI-MS/MS spectra

The extract of culture supernatants from strain TUA-HK1GM cultured in ISP2 medium showed strong antifungal activity against *Aspergillus brasiliensis* but very weak antimicrobial activity against *Staphylococcus epidermidis*. The antifungal fractions were purified through gel filtration and silica gel chromatography to obtain active compound homogeneity on TLC. However, HPLC and ESI-MS revealed that this component contains two active compounds. ESI-MS analysis showed the presence of two molecular ion peaks at *m/z* 813.5128 [M+Na]⁺ and *m/z* 827.4912 [M+Na]⁺ (Fig. 1a). These molecular formulae were determined to be C_45_H_74_O_11_ and C_45_H_72_O_12_, which were consistent with those of oligomycin A and oligomycin B, respectively (their theoretical *m/z* values for [M+Na]⁺ are 813.5123 and 827.4916, respectively) (Fig. 1a). Its ^1^H NMR and ^13^C NMR signals showed good agreement with the reported data for oligomycin A and oligomycin B [19–21] (Figs. S3 and S4). Thus, the potent antifungal compounds produced by strain TUA-HK1GM were determined to be the 26-membered macrolactones oligomycins A and B.

### Strain TUA-HK1GM produces 7-deoxypactamycin in the presence of NaCl

Strain TUA-HK1GM showed a very weak antibacterial effect against *S. epidermidis* when cultured in ISP2 medium. Given that this strain showed higher NaCl tolerance than closely related species, we examined the anti-*Staphylococcus* activity of an extract prepared from the culture supernatant of the strain grown in ISP2-3N medium. The results revealed that the production of anti-*Staphylococcus* compounds was enhanced in the presence of NaCl. The extract of uninoculated ISP2-3N medium was also subjected to the assay and confirmed to be inactive. To identify this antibacterial compound, we purified the active compound extracted from ISP2-3N culture medium using gel filtration and silica gel chromatography and analysed the purified compound using ESI-MS. The ESI-MS analysis showed two molecular ion peaks at *m/z* 543.2813 [M + H]⁺ and *m/z* 565.2633 [M+Na]⁺. Its molecular formula was determined to be C_28_H_38_N_4_O_7_, consistent with 7-deoxypactamycin (its theoretical *m/z* values are 543.2813 for [M + H]⁺ and 565.2632 for [M+Na]⁺, respectively) (Fig. 1b). To confirm that this substance was 7-deoxypactamycin, comparative LC-QToF-MS and ESI-MS/MS analyses were performed with the reference standard 7-deoxypactamycin prepared from a strain of *Streptomyces pactum* ATCC 27456 ^T^ [22]. Standard 7-deoxypactamycin and the purified antibacterial compound were detected at the same retention time in the extracted ion chromatograms at *m/z* 543.28 (Fig. 1c). Identical fragmentation patterns were detected in both the standard 7-deoxypactamycin and the purified antibacterial compound (Fig. 1d), suggesting that 7-deoxypactamycin was produced in the presence of NaCl.

### Genetic characterisation of *olm* and *ptm* BGCs

To investigate the presence of the BGCs responsible for oligomycin and 7-deoxypactamycin biosynthesis in this strain, the genome sequence of strain TUA-HK1GM was analysed using antiSMASH. Annotation of secondary metabolite BGCs revealed the presence of both oligomycin (*olm*) and 7-deoxypactamycin (*ptm*) BGCs. The *olm* BGC contains all the genes required for oligomycin biosynthesis, including type-I PKS genes (*olm*1–*olm*7) as core biosynthetic genes, *olmB*, which encodes a cytochrome P450, and *olmI* and *olmII*, both of which encode transcriptional regulators in the LuxR family. The amino acid sequences of the *olm* BGC in strain HK1GM showed good alignment with those of *Streptomyces avermectinius* ATCC 31267 ^T^ [23] (Fig. S5).

The 7-deoxypactamycin BGC in strain *S. pactum* ATCC 27456 ^T^ includes PKS genes (*ptmI*, *ptmK*, *ptmO*, *ptmQ*, *ptmS*), radical SAM genes (*ptmC*, *ptmH*, *ptmL*, *ptmM*), and genes that encode glycosyltransferase (*ptmJ*) and sugar-modifying enzymes (*ptmA, ptmB, ptmG, and ptmN*) [24, 25]. While the 7-deoxypactamycin BGC in strain TUA-HK1GM also contains these genes, several other genes, *e.g*., *ptmX*, *ptmW*, and *ptmP*, were missing in this strain. Notably, *ptmY*, which shares high homology with the cytochrome P450 monooxygenase gene proposed to be involved in C-7 hydroxylation, was replaced with a transporter gene in strain TUA-HK1GM (Fig. S6).

### NaCl-induced 7-deoxypactamycin and oligomycin production

**Fig. 2:**
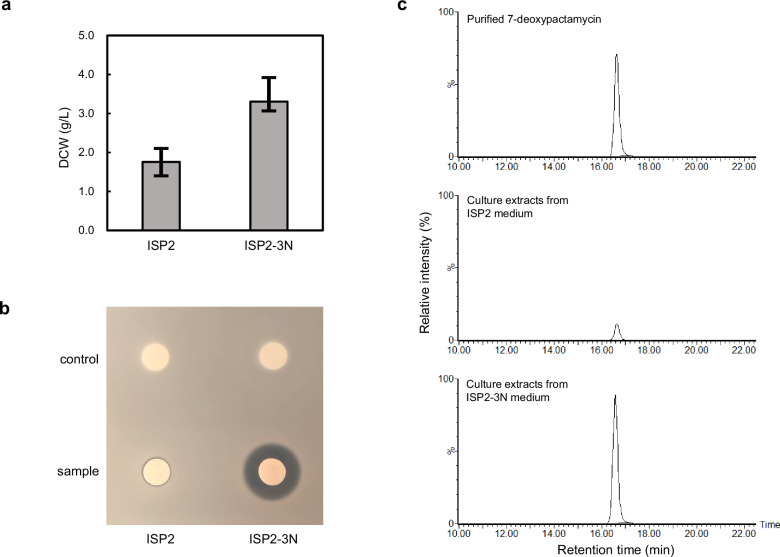
Effect of NaCl addition on 7-deoxypactamycin production. **a** Dry cell weight of strain TUA-HK1GM grown in ISP2 and ISP2-3N media for 10 days. **b** Antimicrobial assay of culture extracts from strain TUA-HK1GM cultured in ISP2 and ISP2-3N media against *S. epidermidis*. Control and sample represent extracts from medium without strain HK1GM inoculation and extracts from medium inoculated with strain HK1GM, respectively. These extracts were dissolved in methanol. **c** Detection of 7-deoxypactamycin in LC-MS. The y-axis represents the relative intensity, where 100% corresponds to an absolute intensity of 1.5×10^7^ counts

To investigate the effect of NaCl on strain TUA-HK1GM growth and 7-deoxypactamycin production, we first measured the dry cell weight (DCW) using ISP2 liquid medium and ISP2-3N liquid medium. Strain TUA-HK1GM exhibited good growth in ISP2-3N medium. The DCW obtained from ISP2-3N medium was approximately twice that obtained from ISP2 medium (Fig. 2a). Given the approximately two-fold difference in DCW, crude extracts from both culture broths were prepared at equal concentrations per unit biomass and subjected to antimicrobial assays. The bioassay revealed that ISP2-3N extract showed a larger inhibition zone against *S. epidermidis* than that from ISP2 culture, indicating enhanced antibiotic activity (Fig. 2b). Both extracts were subjected to LC-QToF-MS analysis and monitored using an extracted ion chromatogram of *m/z* 543.28. 7-Deoxypactamycin was hardly detected in extracts from ISP2 medium without NaCl. However, large amounts of it were detected in extracts from ISP2-3N medium (Fig. 2c).

**Fig. 3:**
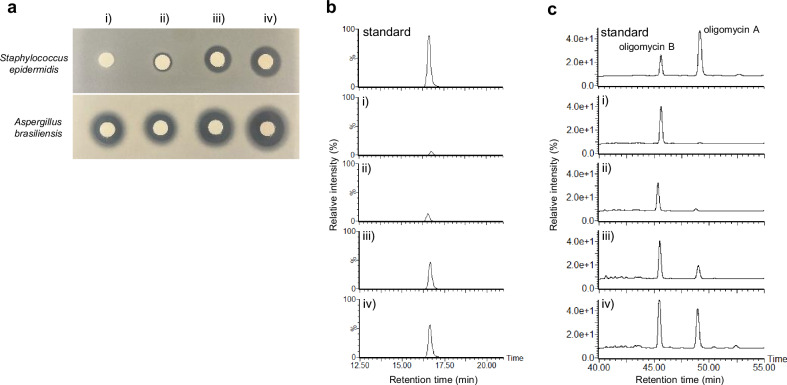
Effect of NaCl addition on 7-deoxypactamycin and oligomycins A and B production. **a** Antimicrobial assay of culture extracts from strain TUA-HK1GM cultured in ⅰ) ISP2, ⅱ) ISP2-3N (exposed to NaCl for 4 days), ⅲ) ISP2-3N (exposed to NaCl for 7 days) and ⅳ) ISP2-3N medium against *S. epidermidis* and *A. brasiliensis*. **b** Changes in 7-deoxypactamycin production due to intermediate supplementation of NaCl. Purified 7-deoxypactamycin as a reference and 7-deoxypactamycin from the culture extract. The y-axis represents relative intensity, where 100% corresponds to an absolute intensity of 1.2 × 10⁷ counts. **c** Changes in oligomycins A and B production due to intermediate supplementation of NaCl. Standard reference of oligomycin mixture and oligomycins A and B from the culture extract. In panels **b**, **c**, (ⅰ) ISP2, (ⅱ) ISP2-3N (exposed to NaCl for 4 days), (ⅲ) ISP2-3N (exposed to NaCl for 7 days), and (ⅳ) ISP2-3N medium (exposed to NaCl for 10 days)

To investigate the effects of NaCl supplementation on antibiotic production in more detail, we assessed 7-deoxypactamycin and oligomycin production by the addition of NaCl at different time points during cultivation. NaCl was added at the start of cultivation in the ISP2 medium (exposed to NaCl for 10 days) or introduced at day 4 (exposed to NaCl for 7 days) or at day 7 (exposed to NaCl for 4 days). Cultures with no NaCl added was used as controls. Under these conditions, bioassay results showed that prolonged exposure to NaCl resulted in larger inhibitory zones against *A. brasiliensis* and *S. epidermidis*, indicating increased antibiotic production (Fig. 3a). Quantification through LC-QToF-MS revealed that the amount of 7-deoxypactamycin detected from culture extracts of ISP2-3N (exposed to NaCl for 4 days), ISP2-3N (exposed to NaCl for 7 days), and ISP2-3N (exposed to NaCl for 10 days) were 2.2-, 8.5-, and 11-fold, respectively, relative to ISP2 (no exposure). These results indicated that 7-deoxypactamycin production was induced by NaCl (Fig. 3b).

The addition of NaCl also affected oligomycin production (Fig. 3c). In the ISP2 medium, oligomycin B was predominantly produced, whereas oligomycin A was scarcely produced. However, the longer the exposure time to NaCl, the more significant the production of oligomycin A. The amount of oligomycin A detected in the culture extracts of ISP2-3N (exposed to NaCl for 4 days), ISP2-3N (exposed to NaCl for 7 days), and ISP2-3N (exposed to NaCl for 10 days) was 1.5-, 10-, and 32-fold relative to ISP2 (no exposure), respectively. For oligomycin B, which is produced regardless of the presence of NaCl, a tendency was also observed for production levels to increase with longer exposure periods to NaCl (Fig. 3c).

## Discussion

In this study, we investigated the phenotypic characteristics and antibiotic productivity of *Streptomyces* sp. strain TUA-HK1GM. Morphological, physiological and biochemical, and phylogenetic analyses revealed that this strain has distinct characteristics that differentiate it from the reference strain *S. griseoruber* DSM 40281 ^T^ [18], including the ability to grow at high NaCl concentrations. Thus, strain TUA-HK1GM is considered a novel species. As this strain showed strong antifungal activity, we first identified the antifungal compounds and determined them to be oligomycins A and B. Oligomycins are 26-membered macrocyclic lactones produced by some *Streptomyces* strains that exhibit a variety of biological activities, including strong antifungal activity by inhibiting mitochondrial FoF1-ATP synthase and immunosuppressive and insecticidal activities [26–30]. Moreover, significant production of 7-deoxypactamycin was observed under NaCl-supplemented conditions. This aminocyclitol antibiotic was first isolated from *Streptomyces* sp. strain SIPI-A3-0121 and subsequently identified in *S. pactum* and other *Streptomyces* strains. It is often co-produced with pactamycin and exhibits antimicrobial and antiprotozoal activities [31–34].

Detailed analysis of the effects of NaCl on antibiotic production revealed that this strain mainly produced oligomycin B in the absence of NaCl. In contrast, NaCl addition activated the production of oligomycin A and 7-deoxypactamycin in addition to oligomycin B. Moreover, the amount of oligomycin B increased upon NaCl addition, even though it was already produced under NaCl-free conditions. To our knowledge, this is the first report of high concentration NaCl can dramatically induce antibiotic production in *Streptomyces*.

The enhanced production of 7-deoxypactamycin may be due to one or more factors, such as increased cellular concentrations of precursors due to NaCl induction, accelerated enzymatic reaction, or activated/deactivated regulators or signalling molecules. The production of oligomycins A and B are also considered to be affected by NaCl-induced acceleration/inhibition of enzymatic reactions or an increase in the supply of their precursors. An example of oligomycin being co-produced with other antibiotics is the co-production of oligomycin and avermectin [35]. In this case, multiple regulatory factors intricately control the biosynthesis of both compounds in a positive or negative manner [36–39]. Meanwhile, in strain TUA-HK1GM, the presence of NaCl was involved in increasing the production levels of both oligomycin and 7-deoxypactamycin, indicating that a co-production activation mechanism different from that reported for oligomycin and other antibiotics is involved. Analysis of chromatograms through LC-MS confirmed that the addition of NaCl also caused increases or decreases, or activation or inactivation of substances other than 7-deoxypactamycin and oligomycins A and B. This indicates that the addition of NaCl exerts a significant effect on secondary metabolism of strain TUA-HK1GM.

The most common isolation source of *Streptomyces* is soil, and a few studies have reported the isolation of this genus from NaCl-containing environments such as seawater, sediments, fishes, molluscs, sponges, seaweeds, and mangroves [3]. However, there are almost no reports of *Streptomyces* isolated from environments with high salinity, antimicrobial activity, or fermented food sources [40]. Furthermore, few studies have examined the antibiotic productivity of *Streptomyces* under NaCl concentrations [41, 42].

Kusaya gravy contains high concentrations of salt, amino acids from raw fish, seawater components, and a complex microbial community with diverse microorganisms. These factors together may generate many microbial metabolites. As this complex and specific environment does not occur naturally, the NaCl-induced antibiotic production of this strain may be adapted to the unique conditions of kusaya gravy. It is also possible that other *Streptomyces* strains in kusaya gravy are similarly capable of thriving in the presence of NaCl and increasing antibiotic production in response to NaCl.

Actinomycetes can produce wide variety of antibiotics, particularly *Streptomyces*, which harbour approximately 20–30 secondary metabolite BGCs. However, many BGCs are not expressed under ordinary laboratory conditions [43, 44]. Thus, silent BGCs must be activated to discover novel antibiotics. In some cases, dormant genes can be expressed using genetic methods, but these requires complex experiments. Induction of antibiotic production by the addition of a simple substance (e.g., NaCl) is an easy yet highly effective method for enhancing and activating antibiotic production.

The detailed mechanism underlying the activation of antibiotic production by NaCl remains unclear. In a future study, we plan to elucidate the mechanism by which NaCl activates antibiotic production in this strain and investigate whether the addition of NaCl also affects antibiotic production in other *Streptomyces* strains isolated from kusaya gravy and other sources. The elucidation of this mechanism is expected to contribute to the discovery of new antibiotics and provide a deeper understanding of the biological significance of antibiotic biosynthesis in Actinomycetes, which is still not fully understood.

## Supplementary information

Supplemental_material
