# Supplementary material for: Salt-induced 7-deoxypactamycin and oligomycin production in Streptomyces sp. strain TUA-HK1GM isolated from kusaya gravy
Source: J Antibiot (Tokyo). 2026 Jun 9;79(8):507–16. doi: 10.1038/s41429-026-00934-y (PMC13402214; doi:10.1038/s41429-026-00934-y)
Supplement: Supplementary file 1 — Supplemental_material [file 41429_2026_934_MOESM1_ESM.docx]

**Supplementary materials**

**Salt-induced 7-deoxypactamycin and oligomycin production in *Streptomyces* sp. strain TUA-HK1GM isolated from kusaya gravy**

Sachiko Masaki ^1^, Sho Ogaki ^2^, Nana Kanayama ^3^, Asahi Hirata ^2^, Aiko Teshima ^2^, Khaled H. Almabruk ^4^, Takahiro Osada ^5^, Ryosuke Unno ^1^, Morio Ishikawa ^1^, Taifo Mahmud ^4^, Kenji Arakawa ^2^ and Toshihiro Suzuki ^1, *^

^1^ Department of Fermentation Science and Technology, Graduate School of Applied Bioscience, Tokyo University of Agriculture, 1-1-1 Sakuragaoka, Setagaya, Tokyo, 156-8502, Japan

^2^ Graduate School of Integrated Science for Life, Hiroshima University, 1-3-1 Kagamiyama, Higashi-Hiroshima, Hiroshima, 739-8530, Japan

^3^ Department of Fermentation Science, Faculty of Applied Bioscience, Tokyo University of Agriculture, 1-1-1 Sakuragaoka, Setagaya, Tokyo, 156-8502, Japan

^4^ Department of Pharmaceutical Sciences, Oregon State University, Corvallis, OR 97331, 541-737-1000, USA

^5^ Osada Shouten, 789 Mitsune, Hachijojima, Tokyo, 100-1511, Japan

^*^Corresponding Author: Toshihiro Suzuki

Email: ts206188@nodai.ac.jp

Mailing address: Tokyo University of Agriculture, 1-1-1 Sakuragaoka, Setagaya, Tokyo, 156-8502, Japan

**Contents**

**Table S1.** Morphological characteristics of strain TUA-HK1GM and *Streptomyces griseoruber* DSM 40281^T^　　　------------------------------------------------　　3

**Table S2.** Physiological and biochemical characteristics of strain TUA-HK1GM and *S. griseoruber* DSM 40281^T^　　　------------------------------------------------　　4

**Table S3.** Genome properties of strain TUA-HK1GM obtained by PacBio Sequel II

------------------------------------------------　　5

**Figure S1.** Morphological characteristics of strain TUA-HK1GM　　　 ------------　　6

**Figure S2.** Tree inferred from Genome BLAST Distance Phylogeny (GBDP) distances using TYGS　　　　　　　 ------------------------------------------------　　7

**Figure S3.** ^1^H NMR spectra of antifungal compounds (500 MHz, CDCl_3_)

------------------------------------------------　　8

**Figure S4.** ^13^C NMR spectra of antifungal compounds (500 MHz, CDCl_3_)

------------------------------------------------　　9

**Figure S5.** Core biosynthetic gene cluster of oligomycin in strain TUA-HK1GM and *Streptomyces avermectinius* ATCC 31267^T^

------------------------------------------------ 　10

**Figure S6.** Core biosynthetic gene cluster of 7-deoxypactamycin in strain

TUA-HK1GM and *Streptomyces pactum* ATCC 27456^T^

------------------------------------------------ 　11

| **Table S1**　Morphological characteristics of strain TUA-HK1GM and *Streptomyces griseoruber* DSM 40281^T^ [18] | | | | | | | |
| --- | --- | --- | --- | --- | --- | --- | --- |
| Medium | Strain TUA-HK1GM | | |  | *S. griseoruber* DSM 40281^T^ | | |
|  | Growth | Aerial mycelium | Substrate mycelium |  | Growth | Aerial mycelium | Substrate mycelium |
| ISP2 | + | grey | grey-brown |  | + | grey | red |
| ISP3 | + | grey | grey |  | + | grey | red |
| ISP4 | + | grey | grey |  | + | grey | red |
| ISP5 | + | grey | grey |  | + | grey | black |

| **Table S2**　Physiological and biochemical characteristics of strain TUA-HK1GM and *S. griseoruber* DSM 40281^T^ [18] | | | |
| --- | --- | --- | --- |
| Tested property |  | Strain TUA-HK1GM | *S. griseoruber*  DSM 40281^T^ |
|  |  |  |  |
| NaCl tolerance (%) |  | 0-7 | 0-5 |
| Melanoid pigment | ISP6 | + | － |
|  | ISP7 | + | + |
| Carbon utilization | Glucose | + | + |
|  | L-Rhamnose | + | + |
|  | D-Mannitol | + | － |
|  | D-Fructose | + | (+) |
|  | L-Arabinose | + | + |
|  | Raffinose | + | － |
|  | Sucrose | + | － |
|  | D-Xylose | + | + |
|  | Inositol | + | + |

+; positive, (+); doubtful utilization, －; negative,

| **Table S3** Genome properties of strain TUA-HK1GM obtained by PacBio Sequel II | |
| --- | --- |
| Genome Statistics |  |
| Total Length (bp) | 11,101,742 |
| No. of sequences | 3 |
| GC content (%) | 71.8 |
| N50 | 10,908,090 |
| No. of CDSs | 9,857 |
| No. of rRNA | 18 |
| No. of tRNA | 90 |
| No. of CRISPERS | 3 |
| Coding Ratio (%) | 89.9 |

**
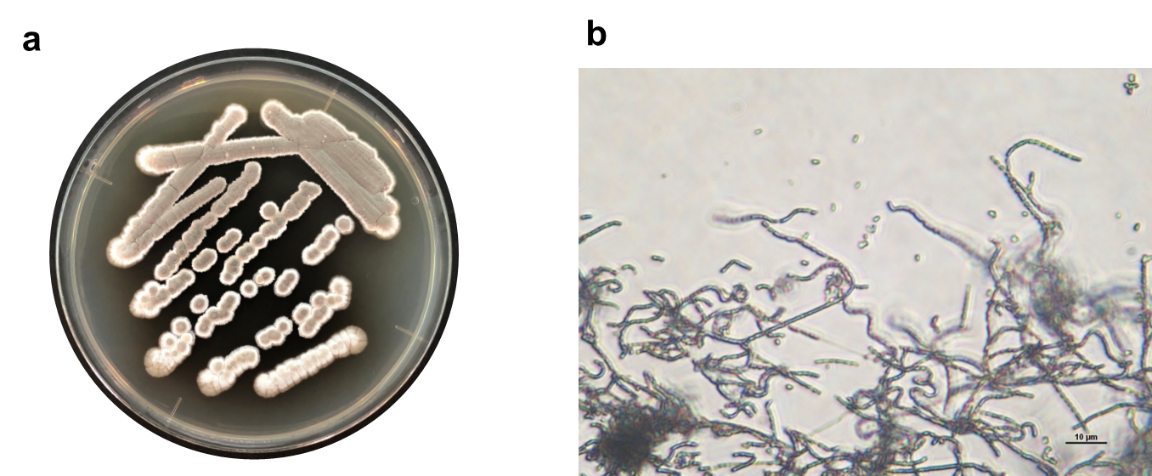
**

**Figure S1** Morphological characteristics of strain TUA-HK1GM. **(a)** Colony on ISP2 agar. **(b)** Spore chain morphology.


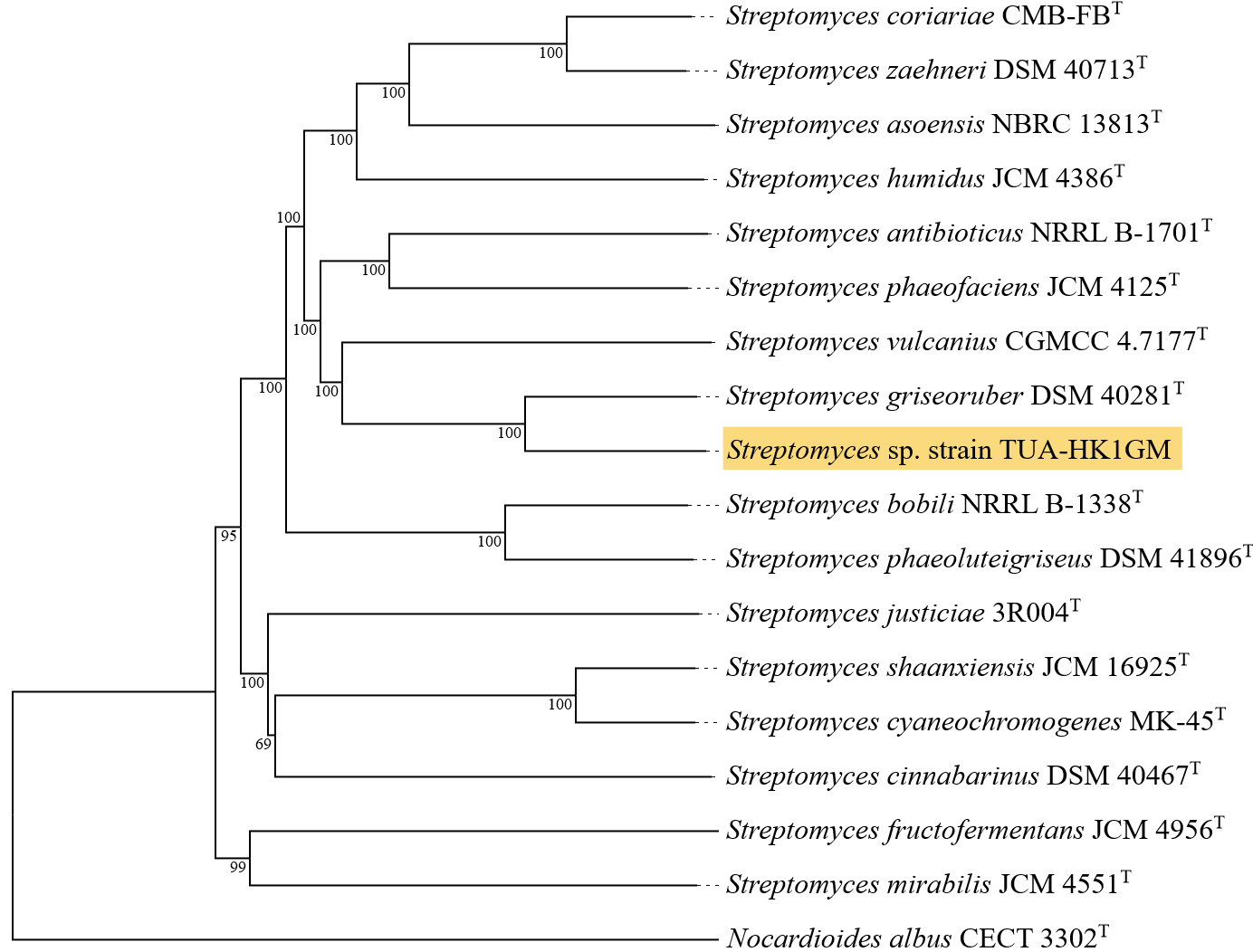


**Figure S2** Tree inferred from Genome BLAST Distance Phylogeny (GBDP) distances calculated from genome sequences using TYGS. The tree was generated using TYGS based on GBDP distances calculated from the genome sequences of strain TUA-HK1GM and related strains deposited in National Center for Biotechnology Information (NCBI) database. *Nocardioides* albus CECT 3302ᵀ served as an outgroup. Numbers at tree nodes indicate bootstrap values based on 100 replicates.

**
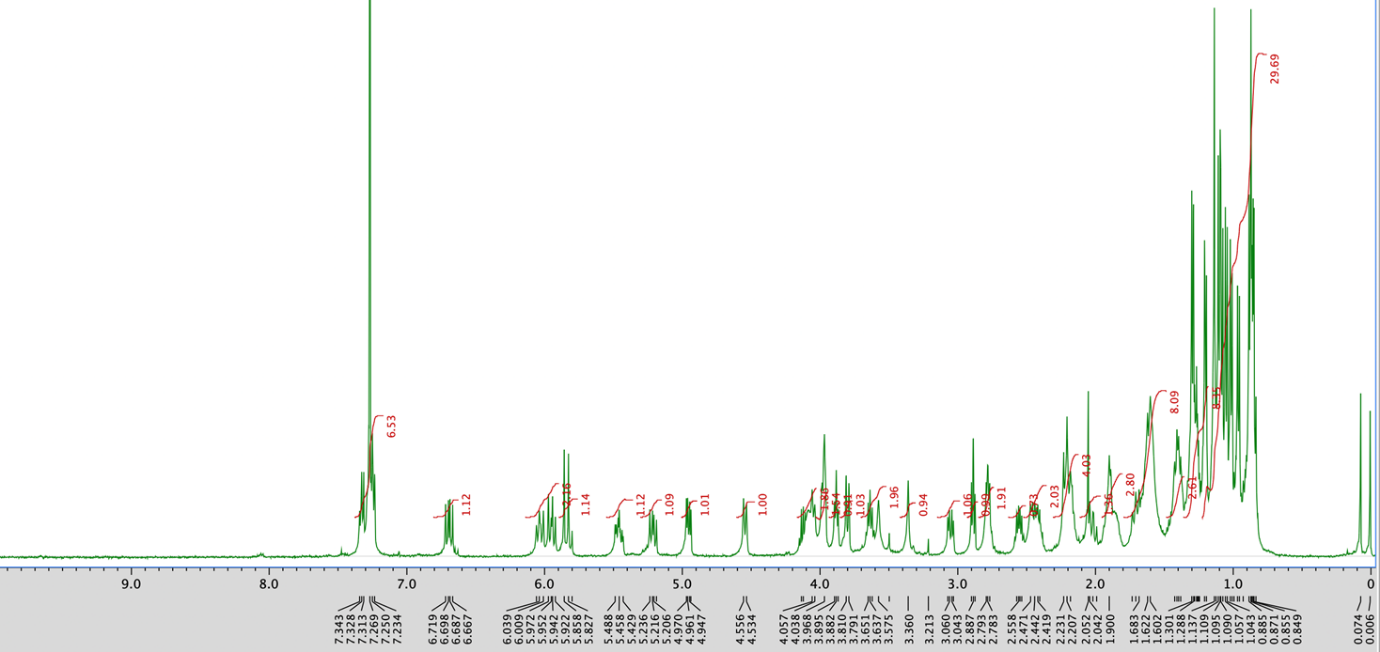
**

**Figure S3** ^1^H NMR spectra of antifungal compounds (500 MHz, CDCl_3_).

**
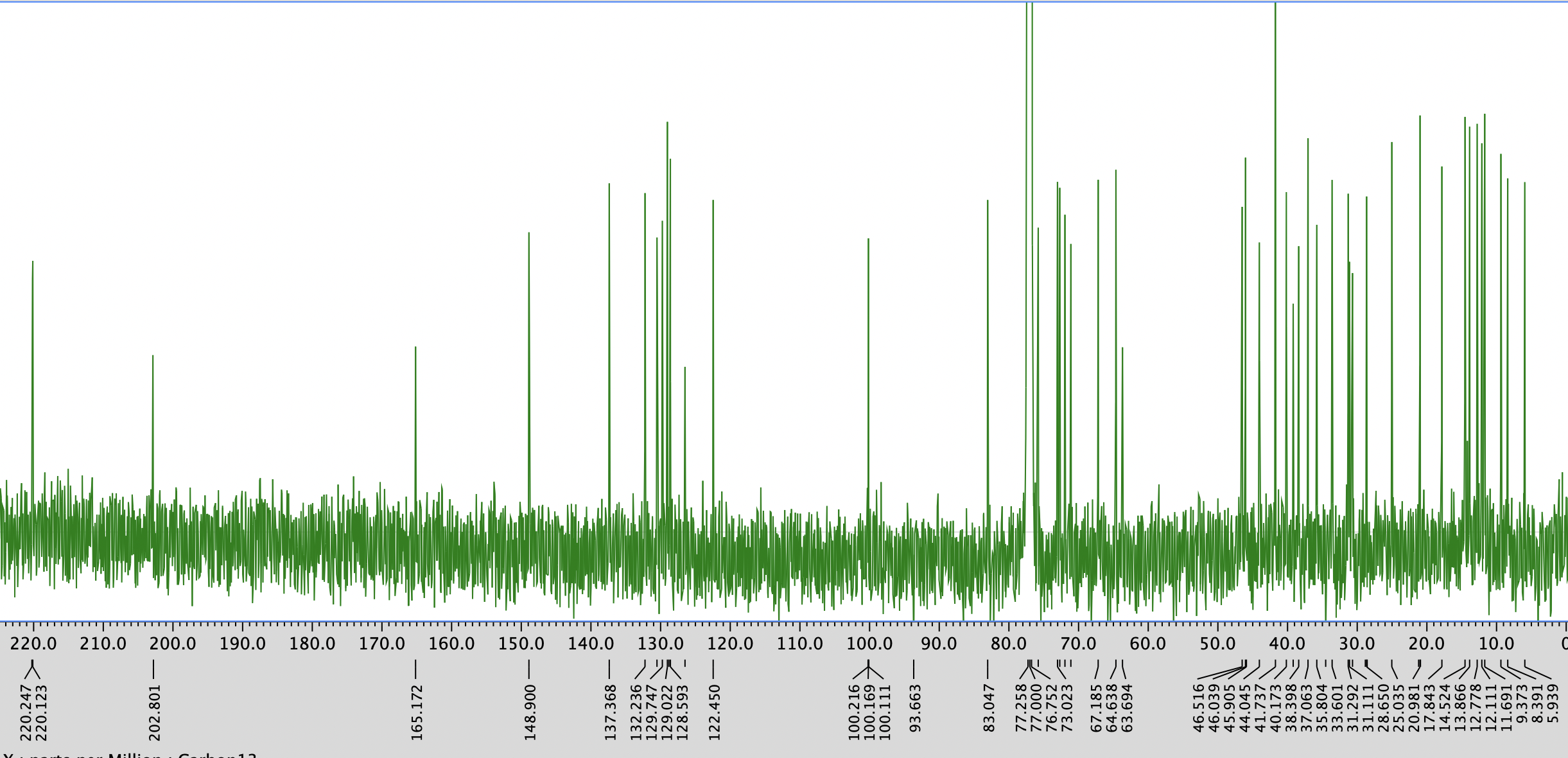
**

**Figure S4** ^13^C NMR spectra of antifungal compounds (500 MHz, CDCl_3_).


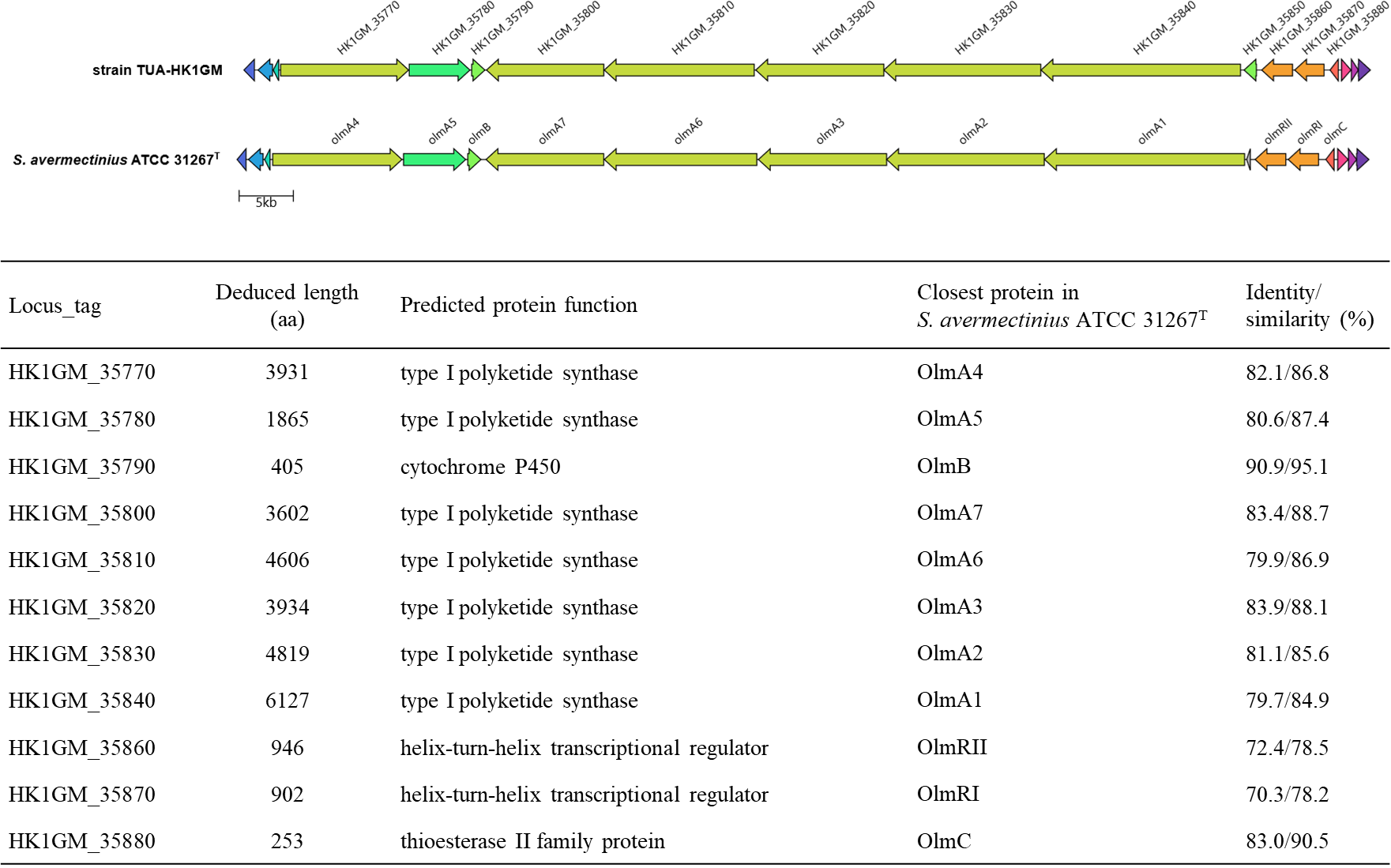


**Figure S5** Core biosynthetic gene cluster of oligomycin in strain TUA-HK1GM and *Streptomyces avermectinius* ATCC 31267^T^. Oligomycin biosynthetic gene clusters in strain TUA-HK1GM are represented under the gene organization. Protein functions were predicted using Blastp (https://blast.ncbi.nlm.nih.gov/Blast.cgi). Amino acid identity and similarity were determined using pairwise alignment algorithm with EMBOSS Needle pairwise sequence alignment tools (https://www.ebi.ac.uk/Tools/psa/emboss_needle/).


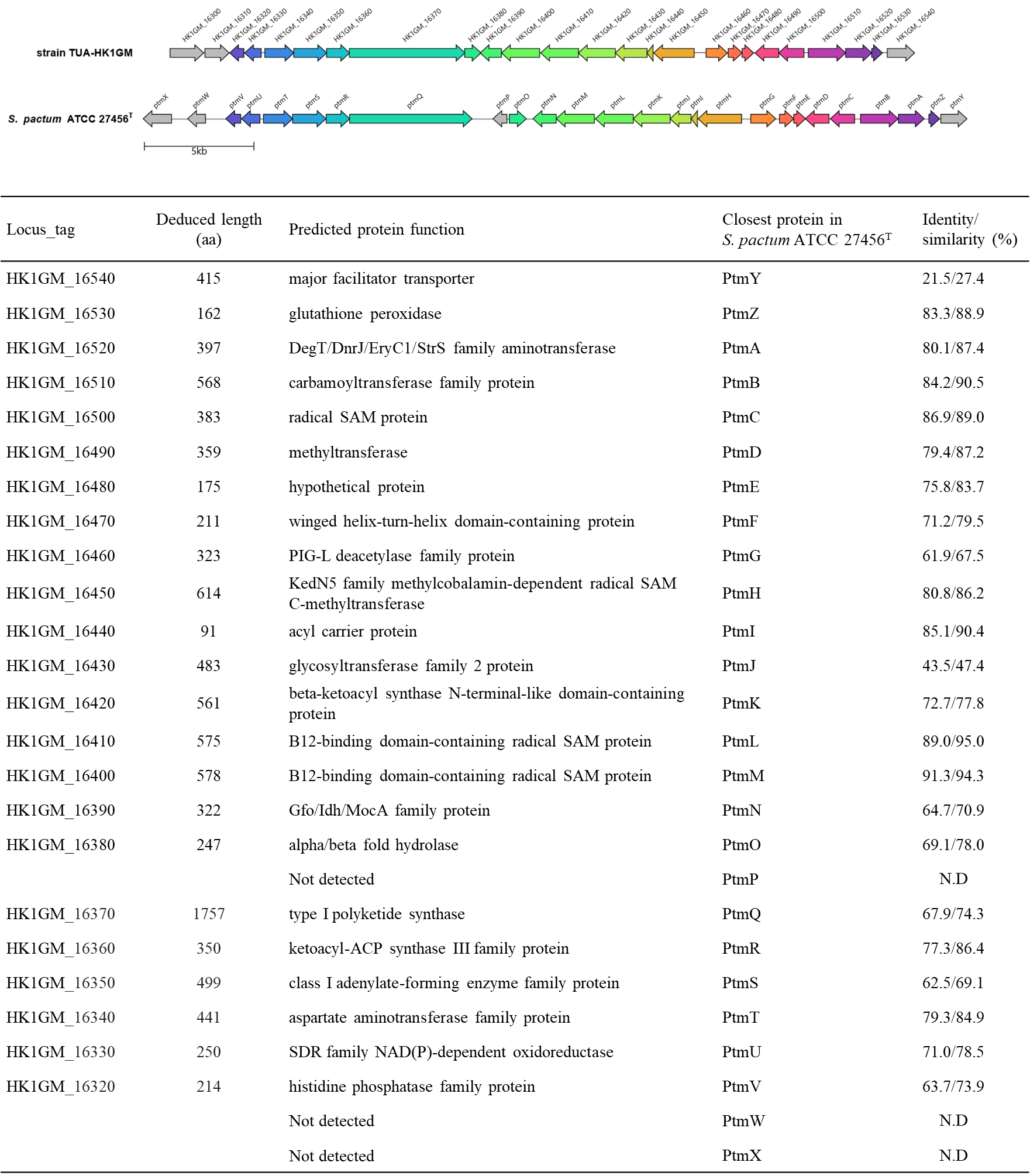


**Figure S6** Core biosynthetic gene cluster of 7-deoxypactamycin in strain TUA-HK1GM and *S. pactum* ATCC 27456^T^. 7-deoxypactamycin biosynthetic gene clusters in strain TUA-HK1GM are represented under the gene organization. Protein functions were predicted using Blastp (https://blast.ncbi.nlm.nih.gov/Blast.cgi). Amino acid identity and similarity were determined using pairwise alignment algorithm with EMBOSS Needle pairwise sequence alignment tools (https://www.ebi.ac.uk/Tools/psa/emboss_needle/).
